# A Year's Work at the Radium Institute

**Published:** 1914-05-30

**Authors:** 


### A YEAR'S WORK AT THE RADIUM INSTITUTE.

With the authority of the committee of management, Mr. Hayward Pinch, the Director of the Radium Institute, has published in the *Lancet* a brief *résumé* of the work done during 1913 by himself and his staff. Wisely, Mr. Pinch for the most part confines himself to statistics and to the relation of his personal experiences and impressions: he is content to let these speak for themselves, rather than to base upon them superstructures of theory and hypothesis which further research may demolish. In essentials, and, indeed, very closely also in details, Mr. Pinch's views are similar to those put forward in the comprehensive account of radium therapy which appeared in our Special Number of March 21. They may be safely regarded as the sanest, most reliable, and most scientific expressions upon radium therapy available to-day for the guidance of medical men, institutional managers, and the public at large.

With a caution truly admirable the Director refuses to label any case of malignant disease as cured, no matter how completely all vestiges of it seem to have evaporated. Nor is this an excess of timidity: it is a timely reminder to a forgetful laity that years must elapse before the risk of recurrence of a malignant tumour can be regarded as eliminated. Purists would say that the chance can never be taken as extinguished; and they would, no doubt, be technically correct, for instances of recurrence after ten, fifteen, or even twenty years of quiescence are on record. But for practical purposes a patient who exhibits no signs or symptoms of morbid growth five years after the disappearance (whether caused by radium, operation, or what not) of a malignant tumour, whose nature has been indisputably demonstrated, may properly be spoken of as "cured." It will thus be some time before the Radium Institute will feel justified in boasting of any "cures." Meanwhile, it is noteworthy that 490 patients suffering from these diseases were treated last year in the Institute, including 111 cases of rodent ulcer. Of the latter, 40 are spoken of as "apparently cured," and 34 more as "improved." Of the remaining 379 (cancer) cases, most of which had been regarded as beyond the resources of surgery, nine only are "apparently cured"; but 141 are improved, and of a good many more the results are not yet noted. Only thirty-six of the whole number of patients have died.

At the present time the popular mind is so excited by newspaper paragraphs concerning radium therapy that a number of unscrupulous swindlers are exploiting it by supplying alleged radium treatment at high prices. We do not say that all such therapeutic agencies are dishonestly conducted, or that all of them are selling substances whose radio-activity exists only in the imagination of the vendors. But we do say this: that for the non-medical public the only sure guarantee against fraud and deception, in a matter which may closely touch the life and the comfort of those dear to them, is provided by the existence of the Radium Institute (or by bodies similarly composed which are being started in a few of the largest provincial cities). Not only is better advice and treatment procurable there than anywhere else; the Radium Institute is also cheaper, for it is not run for profit, and many patients of limited means are treated at rates which no commercially managed business could possibly emulate.

